# Supplementary material for: Impact of crowdfunding, entrepreneurial finance and varieties of entrepreneurial ecosystems after COVID pandemic for rural women
Source: PLoS One. 2026 Jan 22;21(1):e0340966. doi: 10.1371/journal.pone.0340966 (PMC12826522; doi:10.1371/journal.pone.0340966)
Supplement: S1 Table — (DOCX) [file pone.0340966.s001.docx]

**Supplementary Material**

**for**

**Impact of Crowdfunding, Entrepreneurial Finance and Varieties of Entrepreneurial Ecosystems after COVID pandemic for Rural Women**

Zhikang Lyu^1,*^, Natasha Murtaza^2^

^1^Guangdong University of Finance, Guangzhou, 510630, China.

^2^Institute of Agricultural and Resource Economics, Faculty of Social Sciences, University of Agriculture Faisalabad, 38000, Pakistan

^*^Correspondence: [zhikanglyu81@gmail.com](mailto:zhikanglyu81@gmail.com)

**Questionnaire**

Name: _____________________

Age (Years): (15-25), (25-35), (35-45), (45-55),(55-65)

Marital Status: Single/Married/Divorced/Widow

Education (Years): Middle/ Secondary/ Higher

Number of Children: _________

Family System: Nuclear / Joint

**S1Table. Questionnaire**

| **Sr.No.** | **Variables** | **Questions** | **Strongly Agree** | **Somewh-at**  **Agree** | **Neutral** | **Somewhat Disagree** | **Strongly**  **Disagree** |
| --- | --- | --- | --- | --- | --- | --- | --- |
| 1 | Entrepreneurial intention | Do you have proper opportunities to become an entrepreneur? |  |  |  |  |  |
| 2 | Entrepreneurial Intention | Do you have to seek permission and support from male family members? |  |  |  |  |  |
| 3 | Entrepreneurial intention | Do you think you have a Startup Skills? |  |  |  |  |  |
| 4 | Entrepreneurial intention | Do you think you have risk acceptance ability? |  |  |  |  |  |
| 5 | Crowdfunding | Do you think you have proper awareness about crowdfunding? |  |  |  |  |  |
| 6 | Entrepreneurial Finance | Do you think crowdfunding is a financial resource at hand for rural women in Pakistan? |  |  |  |  |  |
| 7 | Crowdfunding | Do you think crowdfunding plays a vital role in the growth of rural women of Pakistan? |  |  |  |  |  |
| 8 | Crowdfunding | Do you think that crowdfunding helps rural women in the future? |  |  |  |  |  |
| 9 | Crowdfunding | Do you think crowdfunding has a positive impact on the entrepreneurial intentions of rural women of Pakistan? |  |  |  |  |  |
| 10 | **Varieties of Entrepreneurial Ecosystems** | Do you think varieties of entrepreneurial ecosystems have a positive impact on the entrepreneurial intentions of rural women of Pakistan? |  |  |  |  |  |
| 11 | Entrepreneurial Finance | Do you think entrepreneurial finance has a positive impact on crowdfunding for rural women in Pakistan? |  |  |  |  |  |
| 12 | Entrepreneurial Finance | Do you think financial drivers or opportunities are available for rural entrepreneurs in order to facilitate them in Pakistan? |  |  |  |  |  |
| 13 | **Social Capital Distribution** | Do you have a proper Social Human Capital? |  |  |  |  |  |
| 14 | **Varieties of Entrepreneurial Ecosystems** | Do you think Product and process innovation can boost the economic growth level? |  |  |  |  |  |
| 15 | **Varieties of Entrepreneurial Ecosystems** | Do you think a High Growth level enhances the economic growth for rural women in Pakistan? |  |  |  |  |  |
| 16 | **Entrepreneurial Finance** | What do you think that there are limited options of alternate financing for example Capital etc.? |  |  |  |  |  |
| 17 | **Social Capital Distribution** | Do you have cultural support as a rural woman in Pakistan? |  |  |  |  |  |
| 18 | **Social Capital Distribution** | Do you think the level of social capital distribution is low for rural women in entrepreneurial ecosystems in Pakistan? |  |  |  |  |  |
| 19 | **Social Capital Distribution** | Do you think Crowdfunding for SMEs is beneficial? |  |  |  |  |  |
| 20 | **Varieties of Entrepreneurial Ecosystems** | Do you think that varieties of entrepreneurial ecosystems have a positive impact on CF for rural women in Pakistan? |  |  |  |  |  |
