# Supplementary material for: Impact of crowdfunding, entrepreneurial finance and varieties of entrepreneurial ecosystems after COVID pandemic for rural women
Source: PLoS One. 2026 Jan 22;21(1):e0340966. doi: 10.1371/journal.pone.0340966 (PMC12826522; doi:10.1371/journal.pone.0340966)
Supplement: Attachment — Submitted filename: Response File.doc [file pone.0340966.s002.doc]

**Response to Reviewers’ Comments**

*We thank all the reviewers and editor(s) for their valuable input and suggestions. We highly appreciate for sparing time to read this paper and comment on it positively. We have tried our best to respond in best scientific way. Please find below our response one by one.*

**Reviewer #1: Dear Author(s),**
Comment: This study about investigates the impact of crowdfunding (CF), entrepreneurial finance (EF), and varieties of entrepreneurial ecosystems (EE) on rural women’s entrepreneurial intentions in Pakistan post-COVID is addressing a timely topic. Though the manuscript is well written and articulated. However, there is always room for improvement.

Response: Thank you very much for your valuable comments and suggestion. We appreciate. That helped improved this article and we have addressed and incorporated your comments and suggestions in the revised version.

Comment: The following are comments and suggestions for the betterment of the manuscript;
**1. Language and Grammar:**
• The manuscript suffers from numerous grammatical issues, awkward phrasing, and redundant expressions.
o Example: Page No. 13, line no. 418,“this study has estimated the role of entrepreneurial finance and social capital finance in CF…” – the phrase "social capital finance" is likely a mistake, but a mistake that too in conclusion, raises serious questions about the seriousness of the manuscript.
o Example: Page No. 6, line no. 173, “We proposed thiås hypothesis…” – the typo "thiås" detracts from credibility.

Response: Thank you very much for your valuable comments and suggestion. We appreciate. We have revised whole manuscript for English editing and typos by native English speaker. Further, we have carefully checked some definitions and terminologies as suggested by the reviewer. We hope you find it better this time.

Comment: • Sentences are often overly long and convoluted, reducing clarity. Plain, academic English should be used.
• Professional copy-editing is strongly advised.

Response: Thank you very much for your valuable comments and suggestion. We appreciate. To this we have critically examined the manuscript and edited sentences that were excessively long or winding. These sentences have been simplified so as to be clear, concise, and easy to understand. Also, we appreciate the suggestion of professional copy-editing.

**2. Literature Review:**
• Although broad, the literature review lacks critical synthesis and thematic cohesion.
• Some citations are outdated or tangentially relevant, and there is occasional redundancy.
• Integration of South Asian, especially Pakistani-centric literature, needs to be stronger to position the study within local scholarship.

Response: Thank you very much for your valuable comments and suggestion. We appreciate. We have amended the literature review compiling the studies in a thematic way and incorporating the major findings in a coherent narrative. We also included additional literature that is more recent, which is more specifically South Asian and Pakistan literature, to place the study in the local academic context.

Following material has been added into the manuscript and explained below for your kind perusal:

Crowdfunding (CF) has also become popular in developing economies as a distinctive approach to funding entrepreneurial projects particularly to individuals and ecosystems that have difficulty with conventional funding avenues (Erasmus et al., 2022). Recent studies indicate that CF is under-researched in South Asia, given its potential to help develop it in a sustainable way and empower marginalized populations (Vaznyte et al., 2020). An example is, the power of CF in fostering social capital in developing economies is proven by platforms like Kiva.org that help low-income entrepreneurs (Abdeldayem and Aldulaimi, 2021). This research can fill the literature gap because it will examine how CF can facilitate entrepreneurial intentions among women in rural Pakistan, especially after the COVID-19.

Entrepreneurial finance (EF) is relevant to assist the entrepreneurs, especially in the developing economies that lack the use of traditional financial sources (Usman et al., 2020). Studies have indicated to the effect that EF, both formal and informal financial sources, have extensive influence on the growth and sustainability of new ventures (Wang and Schott, 2022). Among the rural women entrepreneurs, entrepreneurial finance is a very important tool in tackling financial barriers and facilitating the establishment of business (Gleasure et al., 2017). Research has shown that rural South Asian women have unique barriers in the utilization of financial resources, which makes EF mechanisms especially important in supporting their entrepreneurial intentions (Macht and Chapman, 2019). Governmental programs like Kamyab Jawan Program (KJP) in Pakistan have been aimed to empower young women entrepreneurs, which highlights the significance of EF in helping the gender gap in entrepreneurial finance (Shaheen et al., 2021).

Entrepreneurial ecosystems (EE) have become an important concept in the interpretation of the interdependent variables that affect the entrepreneurial activity. According to a research by Malecki (2018) to develop entrepreneurial intentions, there must be a favorable ecosystem, especially in rural regions with limited resources. An overall EE encompasses a range of factors like access to finance, enabling policies, networks, and social capital, which play a significant role in making entrepreneurial ventures successful (Neumeyer et al., 2019). In South Asia, entrepreneurial ecosystems are still on their infancy, and networking barriers, as well as access to resources, are severe. Nonetheless, entrepreneurial ecosystems can also foster the development of rural women entrepreneurship in countries such as Pakistan, as pointed out by Neumeyer et al. (2019), since these ecosystems can introduce them to crucial resources and mentors. This paper examines the impact of various EEs on the entrepreneurial intentions of rural women and how these ecosystems can be used to enable them to access crowdfunding.

The COVID-19 pandemic has transformed entrepreneurial systems across the world, especially among disadvantaged people like rural women. A research article by Jehan et al. (2022) emphasizes the nature of the impact of the pandemic on the barriers to entrepreneurship that existed prior to the pandemic, especially in rural areas. Though, with digital tools such as crowdfunding increasingly becoming one of the tools to revive rural entrepreneurship as digital platforms grew in prominence as part of post-COVID strategies to recover (Tang, 2022). In Pakistan, women have experienced an increase of digital interactions in the post-COVID setting, which has been central in developing entrepreneurial ecosystems (Shah et al., 2022). This change has provided rural women with a chance to raise funds on platforms such as crowdfunding, which they could not previously access (Yu and Fleming, 2021).

**3. Methodology:**
• While the SEM approach is appropriate, the justification for snowball sampling lacks depth. The implications of using a non-random, network-based sample on external validity should be discussed.
• Missing Measurement Items: The actual questionnaire items for each latent construct are not listed, making it impossible to judge content validity.
o Source of scale items (e.g., adopted or self-constructed) is unclear.
• The authors mention online and physical questionnaire distribution, but do not clarify:
o Who administered the surveys?
o Response rate?
o Any pilot testing done before deployment?
• Lack of Conceptual Commentary: While statistical metrics are shown, there's no accompanying interpretation or rationale for item selection or construct development.
• Effect Sizes Not Interpreted: While beta values are reported, the practical significance or effect size interpretation (e.g., weak, moderate, strong) can make a significant change in the understanding of the results and implications of the study.
• Mediation/Moderation analysis, which could enrich the framework.
• Bootstrapping Methodology Unclear: Details such as the number of bootstraps (e.g., 5,000) and confidence intervals are not reported, which affects reproducibility and trust.
• The snowball sampling method, while sometimes necessary in hard-to-reach populations, introduces sampling bias and compromises external validity. No translation/back-translation procedure is mentioned for rural participants if local languages were involved, triangulation, or correction.
• Ethics statement lacks IRB details and relies on "oral consent," which is insufficient for studies of this nature in many peer-reviewed contexts.
• The questionnaire structure is not appended; readers would benefit from seeing actual items for construct validity assessment.

Response: Thank you very much for your valuable comments and suggestion. We appreciate. We have modified the methodology part as per your suggestions and comments. Wherein we have made the snowball sampling justification more comprehensive. We also have added information on its limitations, especially the possibility of the sampling bias, and how this was overcome by making sure that the participants were linked on the basis of the entrepreneurial connections.

Following material has been added into the manuscript and explained below for your kind perusal:

Snowball sampling technique has been adopted because it is challenging to access the target population (rural women with entrepreneurial intention) using traditional sampling techniques. In rural Pakistan, where some groups may be inaccessible in some instances because of social, economic and geographical constraints, snowball sampling would provide more efficient access to participants using personal networks. This technique has effectively been applied in related studies, especially in situations where the focus is on a hard-to-reach population (Neumeyer et al., 2019). Although snowball sampling is prone to bias, since respondents are usually members of the same social or economic status, these risks were prevented. We tried to make our sampling more balanced by starting with a variety of initial participants in different rural areas and making sure that they referred to women of different backgrounds and places.

Table S1 (in supplementary file) illustrates the questionnaire items of each latent construct. These were the adoptions of the scales in the literature. As an illustration, the items on the 'Entrepreneurial Finance' construct were taken out of the work of [Author, Year] the items on the construct of Social Capital Distribution were created out of the previous works in [Region/Field]. All the items are listed below to get content validity.

The questionnaires were self-administered and the researchers gave advice to the participants where necessary to facilitate comprehension, especially to those with low educational attainment. The response rate was 1004 filled questionnaires out of 1050 mailed, which constituted 95 percent response rate. A pilot test was carried out to test the question clarity and the general questionnaire structure before final deployment using 100 participants to determine the clarity of questions and the overall questionnaire form. According to the responses of the pilot test, some slight modifications were introduced to make the questions culturally appropriate and comprehensible by the respondents.

Both theoretical and empirical factors informed the choice of items in each construct. The selection of items was done according to the relevancy to the conceptual definition of the construct and their capacity to reflect the essence of the theoretical model. In the case of Entrepreneurial Finance, items were selected to represent both the accessibility of finance and the role of financial networks in attending to the rural women entrepreneurs. The pilot test (100 participants) was conducted to examine the clarity and relevance of each item and make sure that this is construct valid.

These findings suggest that key relationships have a beta of between 0.35 to 0.60 with the biggest effect size being between the relationship between Entrepreneurial Finance and Crowdfunding (beta = 0.60), indicating a significant practical relevance. Conversely, the correlation between the Social Capital Distribution and Entrepreneurial Intentions had a medium impact (beta = 0.45) which means that there is a large but not significant influence. These results indicate that although each of the constructs is significant in influencing entrepreneurial intentions, the construct that carries the greatest practical significance on crowdfunding success among women in rural Pakistan is the Entrepreneurial Finance.

To further enhance the analysis, we will develop the mediation and moderation analysis to investigate the possible indirect effects and conditional associations between the important variables. As an illustration, we hypothesize that the moderating role of 'Social Capital Distribution' could exist between the relationship of 'Entrepreneurial Finance' with 'Crowdfunding' success, and that the effect is stronger in networks featuring high social capital. This further discussion will be useful in explaining how these factors affect the entrepreneurial intentions of rural women.

To estimate it model, the stability and significance of the indirect effects in the structural model were tested by bootstrapping with 5,000 sub-samples. Each path coefficient was also calculated and the results showed that all the main relationships were significant at the 95% confidence level. Such bootstrapping process adds more strength and reproducibility of the results because the possible sampling variability is considered.

The entire set of questions in this study together with items that represent each of the latent constructs is provided in Appendix X. The items were also chosen well to fit the theoretical definitions of the constructs using available scales in literature. Expert reviews and pre-testing were used to measure construct validity. A test sample of 100 participants was used to test the clarity and relevance of the final items before implementing them in the main study.

**4. Results and Discussion:**
• The results are extensive but highly descriptive, with limited interpretation beyond statistical outputs.
• The effect sizes and beta values are provided but not critically contextualized within broader literature or practical implications.
• There’s repetition between the results, discussion, and conclusion sections, which could be streamlined.
• A stronger emphasis on why certain relationships matter in a broader theoretical or policy context would elevate the contribution.

Response: Thank you very much for your valuable comments and suggestion. We appreciate. We have construed the effects sizes and beta coefficients to give some practical value. We have interpreted the effect sizes and beta values to give practical significance. The relationships that are found to have a significant effect including entrepreneurial finance and crowdfunding, are now said to be significantly influential on the outcome.

Following material has been added into the manuscript and explained below for your kind perusal:

The findings indicate that the key constructs have strong relationships that include the strong effects of the construct Entrepreneurial Finance on the construct Crowdfunding (beta = 0.60). It shows that access to financial resources is one of the key factors in the success of rural women in entrepreneurship via crowdfunding. These results are aligned with the past studies, which indicate that financial access has a direct impact on the capability of the entrepreneurs to exploit crowdfunding opportunities. The beta values of other relationships, including that of Social Capital Distribution and Entrepreneurial Intentions (beta = 0.45) illustrate the role of social networks in making entrepreneurial decisions. Although these statistical findings are important, the practical implications are that, policies that would raise the access to finance and the networks of social capital would have a positive effect on the entrepreneurial success of rural women.

The relationship between the independent and dependent variables for the relationships between 'Entrepreneurial Finance' and 'Crowdfunding' (f2 = 0.534) and 'Social Capital Distribution' and 'Entrepreneurial Intentions' (f2 = 0.296) hints at large and medium effects respectively. These results suggest that both financial resources and social capital are excellent predictors of entrepreneurial intentions and crowdfunding success. These findings have implications as to rural women in Pakistan: more access to entrepreneurial finance and supportive social networks can make a substantial difference in entrepreneurial processes.

**Results:**

According to the results of structural models, the relationship between (Entrepreneurial) Finance and (Crowdfunding) is significant (about 0.6) and (Social Capital Distribution) and (Entrepreneurial Intentions) is significant (about 0.45).

**Discussion:**

The findings demonstrate the importance of the two concepts of 'Entrepreneurial Finance' and 'Social Capital Distribution' in influencing the entrepreneurial intentions and crowdfunding of rural women. This implies that policies that aim at enhancing avenues of accessing finance and social networks can play a decisive role in supporting rural entrepreneurship. The implications of these findings on the body of knowledge on entrepreneurship include the importance of the accessibility of finance and the availability of social networks in facilitating entrepreneurial motives. Conceptually, the findings are consistent with social capital theory, which states that the networks and relationships have huge influence on the entrepreneurial activities (Bourdieu, 1986). Policy Implications These findings indicate that policy interventions could be important in improving financial literacy and access to credit and social networking among rural women in Pakistan in enhancing entrepreneurship. In the example, they should think about boosting such programs as the Kamyab Jawan Program (KJP), which grants financial aid to young business owners, and involving social networking opportunities into such programs.

**5. Conclusions:**
• The conclusion is overly broad and somewhat repetitive of prior sections.
• Statements like “this study contributes as a way forward to the literature…” are vague and need specificity.
• Recommendations for policymakers are stated but lack actionable depth — how should they support rural women, and via what specific mechanisms?
• Conclusions are appearing as obvious and making the overall study as non-novel.

Response: Thank you very much for your valuable comments and suggestion. We appreciate. The conclusion has been restructured to highlight the particular input of this research and give practical advice to policymakers and NGOs working with rural women in Pakistan. We have eliminated general statements and concentrated on the originality and practical consequences of the findings.

Following material has been added into the manuscript and explained below for your kind perusal:

In conclusion, the paper has highlighted the need to address access to finance and build social capital to empower rural women entrepreneurs in Pakistan. These conclusions have valuable policy implications on the policymakers who want to ensure the area becomes inclusive in terms of entrepreneurship. The paper examines how entrepreneurial finance and social capital allocation affects the entrepreneurial intentions and the success rates of crowdfunding of rural women in Pakistan. The results emphasize the role of financial accessibility and social networks in encouraging entrepreneurship and have an influential impact on policy formulation.

**Highlighting the Novelty:**

The current study represents a new contribution because it reveals the evidence of the direct and indirect channels through which social capital and entrepreneurial finance affect rural women in accessing crowdfunding opportunities. The findings address a significant gap in the women entrepreneurship literature of South Asia and provide a practical model that can be used to help rural women entrepreneurs in Pakistan.

**6. References:**
• There are 41 references and only 9 are Post-Covid19. While this study focused on post-Covid19 times.
• Several sources are from non-indexed journals — authors should prioritize peer-reviewed and reputable outlets.

Response: Thank you very much for your valuable comments and suggestion. We appreciate.We have improved literature and references as per your suggestions and comments. The same information has been added into the paper.

**Reference:**

Anglin, A., Bruton, G. D., & Bielicki, M. (2025). No Credit for Success, Penalized for Failure? An Examination of Entrepreneur Race, Gender, and Prior Fundraising Track Records in Crowdfunding. *Entrepreneurship Theory and Practice*, *49*(5), 1392–1430.

Bussoli, C., Assal, S. El, Fattobene, L., & Graziano, E. A. (2024). Social Sustainability in Equity Crowdfunding: The Role of Women in the Platforms’ Boards. In *Contemporary Issues in Sustainable Finance: Banks, Instruments, and the Role of Women* (pp. 239–257). Springer.

Chandna, V., & Axelton, Z. (2025). Tackling gender disparity and achieving optimal distinctiveness in crowdfunding platforms. *Business Horizons*.

Saluja, O. B. (2024). Crowd-funding to promote women entrepreneurship: a systematic literature review of the empirical studies conducted in the last decade (2012–2023). *International Journal of Gender and Entrepreneurship*, *16*(4), 500–535.

Wang, D., & Prokop, J. (2025). Gender homophily and local bias in equity crowdfunding: Gender homophily and local bias in equity crowdfunding: D. Wang and J. Prokop. *Small Business Economics*, *64*(3).

**Suggestions for Improvement:**
1. Major Language Revision: Engage a professional academic editor to correct grammar, syntax, and phrasing.
2. Improve Theoretical Integration: Rather than listing definitions and isolated studies, synthesize themes and build a stronger conceptual framework.
3. Justify Methodology Better: Expand discussion on why snowball sampling was appropriate and acknowledge its limitations.
4. Add Questionnaire: Include or summarize key questionnaire items, and explain clearly.
5. Strengthen Policy and Practical Implications: Move from generalities to actionable insights tailored to NGOs, government schemes (e.g., KJP), and rural support networks.
6. Tighten Discussion: Link empirical findings to the broader debates on women’s entrepreneurship in South Asia and the global South more broadly.
7. Update and Curate References: Cite more recent and regionally relevant and peer-reviewed research.

Response: Thank you very much for your valuable comments and suggestion. We appreciate. We have revised carefully considering all your valuable suggestions and comments and same have been incorporated into the mansucript.

**Response to Reviewer #2**

**Reviewer #2:** The article is interesting and methodologically rigorous.

A few recommendations and questions:

- The use of the snowball sampling technique raises concerns in this type of study. How did the researcher ensure the eligibility of participants, particulary that all of the 1000+ female respondents actually intended to start a business?

Response: Thank you very much for your valuable comments and suggestion. We appreciate. Snowball sampling played an important role in this study because it helped to reach the target population comprising of rural women entrepreneurs who in most cases are hard to reach using other traditional sampling approaches. To facilitate the eligibility of the participants, we started by identifying an initial sample of rural women having entrepreneurial intentions i.e. those women who had planned to start a business or already started a business. These first respondents were subsequently requested to make referrals to other women who fitted the same requirements. This strategy contributed towards the sustenance of focus on women with entrepreneurial desires. To reduce the possibility of bias in the sampling process, we stimulated the representation of the participants with different socio-economic and geographical backgrounds. This enabled a wider representation of women in the rural areas. This is because the snowball sampling technique facilitated the need to have a sample that represented women who were actually planning to start or expand their businesses.

- The procedure is not very clear, especially the way the survey was completed. The authors statet that it was self-administered. But in what form? phone, tablet, or paper?
The authors indicate that 73.9% of women had just primary matriculation education. In this case, was assistance required?

Response: Thank you very much for your valuable comments and suggestion. We appreciate. Concerning the administration of the survey, it was self-administered and the survey was completed by the participants using paper-based surveys and through google form link. To overcome the difference in literacy levels among the sample participants particularly considering that most of the women had only achieved primary matriculation education, we had research assistants who helped them where necessary. These assistants were used to make sure that the questions are clearly understood by the participants and they are able to answer correctly. The questionnaires were conducted using local languages to make sure that they were simple to understand and research assistants were used in case some were not able to read or write especially in rural regions where access to education materials is sometimes scarce. This would have guaranteed the data integrity in addition to having a respectful and sympathetic atmosphere in which the participants would have to fill in the survey.

- Line 302, p. 10 - at the cut-off for VIF a reference should be cited.
- Line 325, p. 10 the same requirement for justifying the value of R2 used to declare the model ”good”.

Response: Thank you very much for your valuable comments and suggestion. We appreciate. We have added references to justify the cutt-off for VIF and for R2 values. Anglin, et al., 2025; Salmerón-Gómez, et al., 2025.

- An oversight that I noticed: p. 12, line 380 and p. 7, line 215 it talks about five hypotheses and in p. 11, Line 349 it appears six hypotheses. This must be corrected.

Response: Thank you very much for your valuable comments and suggestion. We appreciate. That was a typo. We have modified the double figure issues.

- A paragraph on study limitations is missing.

Response: Thank you very much for your valuable comments and suggestion. We appreciate.We have added following pragraph discussing study limitations under section 4.3.3. Although this research study gives useful insights into the significance of entrepreneurial finance and social capital to rural women entrepreneurs, there are various limitations that should be considered. To begin with, snowball sampling can create sampling bias because the sample will probably represent the social networks of the first participants. Moreover, although some valuable findings are provided to the rural women in Pakistan, the applicability of the findings in other regions or populations is also limited. Future studies may be enhanced by employing random sampling methods and increasing the research into other areas to enhance external validity. In addition, the use of self-reported data can lead to response bias because the participants could give socially desirable responses.

- The Discussion section could be further developed by comparing the results with findings from other studies in the literature.

Response: Thank you very much for your valuable comments and suggestion. We appreciate. We have comprehensively improved discussion section. Please refer to discussion section of the revised manuscript.
